# Cotrimoxazole Induced Steven Johnson Syndrome: A Case Report

**DOI:** 10.31729/jnma.4999

**Published:** 2020-09-30

**Authors:** Ayushma Acharya, Shreedhar Prasad Acharya, Tulsi Ram Bhattarai

**Affiliations:** 1 Kathmandu Medical College, Sinamagal, Kathmandu, Nepal; 2 Department of Internal Medicine, Kathmandu Medical College, Sinamagal, Kathmandu, Nepal

**Keywords:** *cotrimoxazole*, *pneumonia*, *Steven Johnson syndrome*

## Abstract

Steven Johnson syndrome and toxic epidermal necrolysis are severe and rare adverse drug reactions usually caused by drugs like antiepileptics, penicillin and allopurinol and sometimes also due to infections, malignancy or idiopathic in some cases. Here we are reporting a case of a 50 years female who came with complaint of a burning sensation on the upper half of the body with atypical flat target lesion that later coalesced involving her face, chest and bilateral upper limbs. On examination, positive nikolsky sign and tenderness with <10% body surface area involvement was noticed. The diagnosis of cotrimoxazole induced Steven Johnson syndrome was made. Patient was shifted to intensive care unit and given supportive care along with prophylactic teicoplanin, itraconazole and dexamethasone. The mechanism of eruptions in our patient was due to cotrimoxazole. Cotrimoxazole induced Steven Johnson syndrome is rare and the supportive management with broad spectrum antibiotic and the corticosteroid was enough to beat this life-threatening condition.

## INTRODUCTION

Steven Johnson Syndrome (SJS) and Toxic Epidermal Necrolysis (TEN) are considered as a single spectrum of disease, characterized by detachment of dead epidermis and erosion of mucous membrane along with a positive Nikolsky sign.^1^ It is a type 4 hypersensitivity reaction with release of various cytotoxic signals activated by cytotoxic T lymphocytes and natural killer cells.^2^ SJS and TEN are separated based on body surface area with Steven Johnson syndrome having <10% and toxic epidermal necrolysis having >30% involvement.^1^ A study by Yang et al had reported that the incidence of SJS and TEN to be 1.0 to 6.0 per million and 0.4 to 1.2 per million, respectively but this is 2 fold for the Asian population.^2^ Although uncommon they are associated with high mortality.^3^

## CASE REPORT

**Figure 1:**
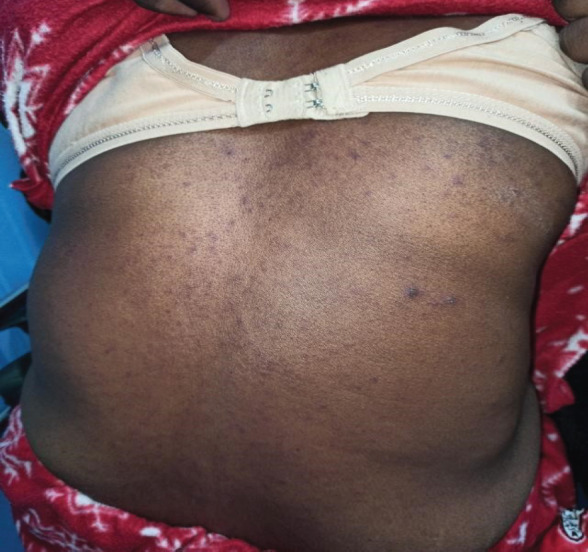
Purpuric macules and papules with atypical target on the posterior aspect of trunk.

A 50-year-old female came to our hospital with a chief complaint of burning sensation over the upper half of her body with development of atypical reddish-purple target macules and papules over her chest, neck and face since 1 day. The lesions initially pin head in size later coalesced to form patches of atypical target lesion over bilateral upper limbs and chest symmetrically. The lesion was also itchy. Few scattered lesions were present in lower limbs as well. The lesions also appeared in the lips early the following morning with patient having symptoms of difficulty in swallowing and watering of eyes. Patient was under cotrimoxazole for 2 weeks due to a history of unresolving pneumonia and recent wound excision procedure 2 months back. Patient left the drug only 3 days back. Patient was a diabetic under metformin and linagliptin since the past 4 years and hypertensive on losartan since the past 2 years. Patient was tachycardic. On examination, patient had multiple purpuric to atypical targetoid macules and patches over the face, chest, neck, upper limbs and trunk ranging from 0.3 × 0.3 cm^2^ to 3 × 3 cm^2^ (Figure 1).

**Figure 2:**
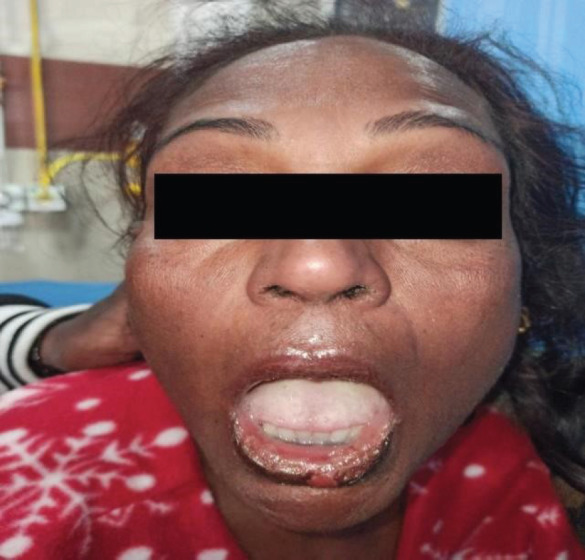
Hemorrhagic crust with erosion of mucosa of lips.

Multiple erosions were present over her lips with yellowish and hemorrhagic crust (Figure 2). Congestion of both eyes with a symblepharon of the left eye was present. Whitish plaque over the dorsum of the tongue was present. Genitalia was spared. Nikolsky sign was positive, tenderness was positive and <10% BSA was involved. The prognosis assessing SCORTEN score was 4 at the time of admission. On investigation, hemoglobin level was 8.2gm% and 24 hours urine protein was 1904.5 mg which was significantly high. Blood smear showed normocytic normochromic anemia, with leukopenia and reticulocyte level of 0.7%. Patient was shifted to ICU due to deranged renal function and decrease in hemoglobin concentration.

**Figure 3A 3B:**
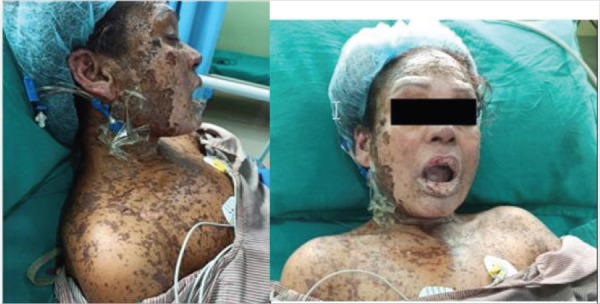
Hyperpigmented macules to patches with areas of desquamation after 2 weeks of disease (A), Red to yellow crust with areas of hemorrhage in mucosa of lips with re-epithelization of face (B).

Patient was treated with intravenous (iv) teicoplanin 200mg for 10 days, iv dexamethasone tapering dose for 12 days, along with magnesium supplementation and 1 pint PCV transfusion. Patient was kept on an insulin sliding scale due to her diabetic history and high blood sugar. Topical anesthetic mucopain and fusid-b (betamethasone) were used in oral mucosa. Ciprofloxacin eye drops and refresh tears were used in the eye and symblepharon was removed. Aloe Vera gel, Vaseline jelly and cloderm were used on skin lesions topically. There was progressive denudation of skin after blistering and the patient started re-epithelization at about two weeks (Figure 3A,4A). Patient did not have any wound swab culture positive and recovered steadily. Patient was discharged after 17 days of admission on oral prednisolone, candid mouth paint and moisturizing aloe Vera cream.

## DISCUSSION

The first reported case of Steven Johnson syndrome was published in 1922.^4^ Though drug related eruptive rashes were noted, the exact etiology of disease was not known. The usual etiology of SJS-TEN is mostly a drug with or without a combination of infection and sometimes idiopathic and malignancy-related causes.^5^ The disease usually begins between 4 and 28 days after culprit drug administration and are rare with drugs administered more than 1 month back.^1^

Our patient was under cotrimoxazole for a duration of 2 weeks, along with features of unresolving pneumonia which could very well be the contributing factor. Among the high-risk drugs that cause the disease are penicillin as the most common antibiotic, carbamazepine as the most common antiepileptic and allopurinol in Asian population.^2^ There are some incidences of cotrimoxazole induced SJS although it doesn't come under the most popular drugs accredited to cause the disease.^6^ However strong temporal relation between administration of cotrimoxazole and appearance of cutaneous eruptions have been established.^7^ The risk of development of SJS with cotrimoxazole is one per year per million population.^8^

Usually patient present with features of viral prodrome like fever and flu like illness.^3^ Our patient did have watery eyes but no typical features of flu like illness were present. Our patient had some of the poor prognosis factors like hyperglycemia, elevated urea and decreased bicarbonate that was used to assess mortality using severity of illness score of toxic epidermal necrolysis scale (SCROTEN).^9^

The treatment of our patient included intravenous and topical steroid along, iv fluid and regular dressing. Teicoplanin was used as broad spectrum antibiotic as there is a risk of drug eruptions with vancomycin as well which is a good prophylactic for gram positive organism.^10^ However there is no enough evidence that any specific treatment beside supportive care is of benefit for patients with SJS-TEN.^1^

## Consent:

**JNMA Case Report Consent Form** was signed by the patient and the original article is attached with the patient's chart.

## Conflict of Interest

**None.**
